# Using Bacterial Extract along with Differential Gene Expression in *Acropora millepora* Larvae to Decouple the Processes of Attachment and Metamorphosis

**DOI:** 10.1371/journal.pone.0037774

**Published:** 2012-05-24

**Authors:** Nachshon Siboni, David Abrego, Francois Seneca, Cherie A. Motti, Nikos Andreakis, Jan Tebben, Linda L. Blackall, Tilmann Harder

**Affiliations:** 1 Australian Institute of Marine Science, Townsville, Australia; 2 School of Biological, Earth and Environmental Sciences and Centre for Marine Bio-Innovation, University of New South Wales, Sydney, Australia; J. Craig Venter Institute, United States of America

## Abstract

Biofilms of the bacterium *Pseudoalteromonas* induce metamorphosis of acroporid coral larvae. The bacterial metabolite tetrabromopyrrole (TBP), isolated from an extract of *Pseudoalteromonas* sp. associated with the crustose coralline alga (CCA) *Neogoniolithon fosliei*, induced coral larval metamorphosis (100%) with little or no attachment (0–2%). To better understand the molecular events and mechanisms underpinning the induction of *Acropora millepora* larval metamorphosis, including cell proliferation, apoptosis, differentiation, migration, adhesion and biomineralisation, two novel coral gene expression assays were implemented. These involved the use of reverse-transcriptase quantitative PCR (RT-qPCR) and employed 47 genes of interest (GOI), selected based on putative roles in the processes of settlement and metamorphosis. Substantial differences in transcriptomic responses of GOI were detected following incubation of *A. millepora* larvae with a threshold concentration and 10-fold elevated concentration of TBP-containing extracts of *Pseudoalteromonas* sp. The notable and relatively abrupt changes of the larval body structure during metamorphosis correlated, at the molecular level, with significant differences (*p<0.05*) in gene expression profiles of 24 GOI, 12 hours post exposure. Fourteen of those GOI also presented differences in expression (*p<0.05*) following exposure to the threshold concentration of bacterial TBP-containing extract. The specificity of the bacterial TBP-containing extract to induce the metamorphic stage in *A. millepora* larvae without attachment, using a robust, low cost, accurate, ecologically relevant and highly reproducible RT-qPCR assay, allowed partially decoupling of the transcriptomic processes of attachment and metamorphosis. The bacterial TBP-containing extract provided a unique opportunity to monitor the regulation of genes exclusively involved in the process of metamorphosis, contrasting previous gene expression studies that utilized cues, such as crustose coralline algae, biofilms or with GLW-amide neuropeptides that stimulate the entire onset of larval metamorphosis and attachment.

## Introduction

**Figure 1:**
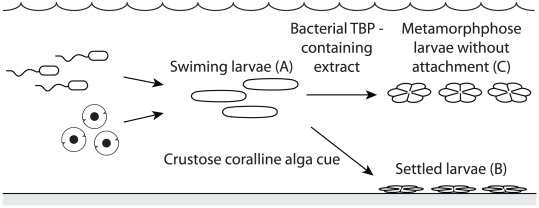
Schematic of coral larval behaviour following exposure to CCA or the bacterial TBP-containing extract. Swimming larvae (A) settle (attach and metamorphose) when exposed to CCA (B) and metamorphose without attachment when exposed to bacterial TBP-containing extract (C).

Attachment and metamorphosis of coral larvae are essential processes for the de-novo colonization and rejuvenation of coral reefs. Coral larvae rely on habitat-specific cues to identify a substratum for successful attachment and metamorphosis, hereafter defined as settlement (Figure 1B). The surface-associated microbial biofilm, characterized by its community composition, abundance and structure, as well as and physical properties of the substratum have been identified as critical factors involved in the induction of settlement of a wide range of marine invertebrate larvae [1]–[4]. In aquaria lacking suitable surface settlement cues, coral larvae have been maintained for months without attachment or metamorphosis (Figure 1A) [5], indicating the crucial role larval settlement cues play in the life cycle of corals.

In a variety of marine invertebrates the community composition and abundance of surface-associated bacteria determine the magnitude of larval settlement [3]. Among these bacteria, γ-proteobacteria of the genus *Pseudoalteromonas* have been shown to be very potent inducers of larval settlement and/or metamorphosis in phylogenetically diverse species such as corals [6]–[8], sea urchins [9] and polychaetes [10]–[12]. We [6], [7] have previously demonstrated that *Pseudoalteromonas* strain A3 [AF313497] associated with the surface of the crustose coralline alga (CCA) *Hydrolithon onkodes* [6], and *Pseudoalteromonas* strains J010 [JF309049], J021 [JF314511] and J104 [JF314512] associated with the surface of the CCA *Neogoniolithon fosliei* [7] induced larval metamorphosis of acroporid coral larvae. We have isolated, identified and confirmed the inductive bacterial metabolite as tetrabromopyrrole (TBP), which is produced by all four strains [7]. While crude extracts of these *Pseudoalteromonas* strains induced high levels of larval metamorphosis, only a small percentage (0–2%) of larvae firmly attached to the substratum, indicating that TBP bypassed substratum-attachment and directly triggered rapid metamorphosis (Figure 1C). This is analogous to the response of acroporid coral larvae to GLW-amide neuropeptides [13], which induce nearly 100% metamorphosis [14], but with markedly higher percentages of attachment (40–80%) [14]. The selective induction of larval metamorphosis by TBP provides a unique opportunity to study gene regulation during this specific developmental event and to decouple the transcriptomic processes of larval metamorphosis from attachment, something which has not previously been achieved with the use of CCA [15]–[17], biofilms [1], [3], nor with GLW-amide neuropeptides [16], [17].

Settlement of marine invertebrates is considered to be a common evolutionary trait among phylogenetically unrelated taxa [18], however the molecular processes enabling the transition of swimming larvae into sessile juveniles are poorly understood. Recent studies have focused on the response of larvae to inducers of settlement at the molecular level [15]–[17], [19]–[21]. A range of genes, including immunity-related genes potentially involved in ontogenetic processes such as attachment, metamorphosis, calcification and symbiosis, have been identified in corals [15]–[17], [19]–[21]. Furthermore, a considerable number of genes were found to be directly involved and/or display differential expression levels in response to external factors such as pathogens and environmental stressors that may interfere with larval settlement [15]–[17], [22]–[26].

The genetic response patterns of marine invertebrate larvae to settlement cues are complex and potentially multi-levelled, and the distinction of specific genetic responses from more generic ones is highly challenging. For instance, in the ascidian *Boltenia villosa*, lectins associated with the innate immune system are rapidly activated after detection of specific settlement cues that ultimately result in metamorphosis [27]. Lectins are also differentially expressed during metamorphosis in the reef building coral *A. millepora* [15]–[17]. Fluorescent protein genes have also been shown to be down-regulated in *A. millepora* following thermal stress [28], larval attachment and metamorphosis [29]. The identification of these genes is facilitated by the rapidly expanding volume of publically available expressed sequence tags (ESTs) and genome-scale microarray screenings in *A. millepora* [15], [16]. Moreover, annotation of new protein sequences is supported by the fully sequenced genomes of the basal cnidarians *Hydra magnipapillata* [30], and *Nematostella vectensis* [31] as well as the recently released genomes of *Acropora digitifera* [32] and *A. millepora* (http://coralbase.org/). The use of these data in molecular techniques, such as microarrays [15], [16], [28] and RNA-sequencing [17], bear high potential as expression profiles of specific genes can be related to critical biological processes (e.g. larval development, immunity, settlement). These molecular techniques, reviewed in Souter et al. [26], are capable of inferring gene expression profiles from thousands to millions of mRNAs. Given the knowledge from a genome-scale gene expression analysis, alternative approaches such as the gene expression profiling technique reverse-transcriptase quantitative polymerase chain reaction (RT-qPCR) [26] can specifically target a select number of genes corresponding to previously identified or putative bio-markers amongst the thousands of genes and monitor their expression in response to a biological process at the population level. This procedure boasts improved accuracy, higher sensitivity, reduced labor and lower cost than single gene quantitative real time PCR screening and microarray gene expression surveys.

Presented here is the application of two multiplex RT–qPCR assays capable of profiling expression levels from a total of 52 selected genes in *A. millepora*, including 47 genes of interest (GOI) and 5 reference genes (RG). The GOI were selected based on their putative role(s) in signalling and the regulation of the processes of settlement and/or metamorphosis (Table 1), including cell proliferation, apoptosis, differentiation, migration, adhesion and biomineralisation. Some of the selected genes have also been implicated in the coral's innate immune defence pathways against pathogens and environmental stressors. Several GOI belong to the MAPK signalling pathway, the NF-κB signalling pathways, or the TIR domain signalling cascade, all of which play major roles in host responses to microbial infections. Genes that are taxonomically restricted genes and are required for coral metamorphosis or calcification, particularly in the early stages of exploration and attachment have also been included (see tables S1 and S2).

**Table 1:** Gene types and their functions associated with marine invertebrate larval settlement and metamorphosis.

Genes	Function associated with settlement and metamorphosis
Apextrin	Extracellular protein is involved in apical cell adhesion and has been shown to be up-regulated during metamorphosis in sea urchins [40], [41] and during *A. millepora* settlement with similar pattern to those of the CEL-III lectins [15], [17].
Tx60-A	Venom gene expressed in Hydra. Related gene has also been identified in *A. millepora* EST dataset. [19].
Septin	Shown to be involved in larval attachment and metamorphosis of *Hydroides elegans* [50].
LRR	Toll/TLR pathway. Provides a versatile structural framework for the formation of protein-protein interactions and responsible for pattern recognition in *Hydra magnipapillata* [58].
cAMP	Toll/TLR pathway. Up-regulated during metamorphosis in a variety of invertebrates [44], [52], [59].
*tyr-amTase*	Tyrosine aminotransferase signal transduction gene in *Haliotis asinina* was down-regulated in post larvae induced by *Hydrolithon onkodes* [51], [60].
GABA	*Haliotis rufescens* larvae induced to settlement and metamorphosis in response to GABA-mimetic peptides [43]–[45].
NADH	Highly regulated metabolic gene during settlement and metamorphosis of Hydroides elegans, and in *Haliotis asinine* following induction by the CCA *Mastophora pacifica* [22], [51].
Lectins (lectin, millectin, CTL, HL)	Implicated in cell recognition and lysis for tissue remodelling during settlement and metamorphosis and mediates symbiont engagement and maintenance of zooxanthellae–coral symbiosis [15], [16], [23], [39].
Pha-C3	cAMP-dependent protein kinase A mediates larval development and settlement in *Hydroides elegans* with increased expression during larval development [52].
Fluorescent proteins	Moderate down-regulation of transcription of GFP-like proteins has been previously associated with attachment and metamorphosis in *A. millepora* larvae [29], [61].
*Amgalaxin-like-1*	A calcium-handling protein expressed strongly in pre-settlement planulae and primary polyps during attachment and metamorphosis [20].
SCRiPs	Taxonomically restricted to Scleractinia. Changes in expression patterns at different life stages including pre and post-settlement, suggesting a distinct role in coral development [21].
Actin	Variations in expression levels during attachment and metamorphosis of the bryozoan *B. neritina* [38], [62].
Integrins	Metazoan receptors for cell adhesion. Shown to provide a connection between the extracellular matrix and the intracellular actin cytoskeleton [63], [64].

The ability of bacterial TBP-containing extract to induce coral larval metamorphosis with little or no attachment was used to decouple the transcriptomic processes of attachment and metamorphosis. The differential gene expression response of *A. millepora* larvae exposed to bacterial TBP-containing extract was investigated using RT-qPCR. Our findings are described below in the context of larval metamorphosis.

## Results

### Assessment of ontogenetic larval development

Coral larvae were exposed to either a threshold concentration (3.5 ng/ml) or to a 10-fold elevated concentration (35 ng/ml) of the bacterial TBP-containing extract. Six hours post exposure, only swimming larvae were observed in treatments and controls (Figure 1A). Twelve hour post exposure, larvae displayed typical signs of metamorphosis but without attachment (i.e. larvae appeared as flattened discs and displayed obvious septal mesenteries radiating from the central mouth region; Figure 1C), whereas larvae in the control treatment were elongated and actively swimming (Figure 1A).

Of the 20 GOI included in Assay 1 (Table S1) and the 27 in Assay 2 (Table S2) all but five (*TRAM*, *HL-1* and *HL-3*; and *tyr-amTase* and *Integrin-alpha1*, respectively) resulted in measurable gene amplification compared to the negative control and were thus included in further analyses and discussed. GOI that did not amplify and/or had >25% coefficient of variation (CV) among technical replicates were removed from further analyses. Following a stepwise exclusion process, data analysis using GeNorm (http://medgen.ugent.be/genorm/) [33] identified *GAPDH* and *MARK-p38* (Assay 1), and *cAMP/ATF4* and *LRRC59* (Assay 2) as the most stable genes demonstrating relatively constant expression levels throughout the different experimental treatments. All expression levels were subsequently normalized against geomean (Excel 7) calculated from these stable genes. The average percentage of CV in technical replicates was 6.5% (Assay 1) and 8.6% (Assay 2). The negative controls did not produce any measureable PCR products confirming that electropherograms resulting from the larval samples reflected transcribed gene amplification products.

### Assay 1

**Figure 2:**
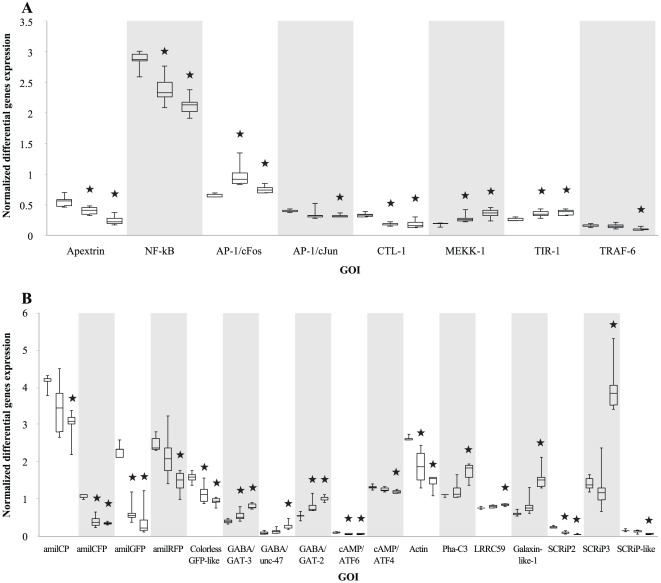
Expression levels of GOI following exposure of *A. millepora* larvae to the bacterial TBP-containing extract. (A) Assay 1 and (B) Assay 2. The left box plot for each gene represents swimming larvae exposed to control (no extract), the middle and right box plots represent swimming larvae exposed to low and high concentration treatments, respectively. The box plots denote GOI that presented significant differences in expression (*p<0.05*, Kruskal–Wallis followed by multiple comparison of mean ranks) from the control in one or both of the treatments. The gene expression levels were normalized against geomean (Excel 7) calculated from the most stable genes. An asterisk (★) highlights those GOI that were significantly different from the control.

Twelve hours post exposure of coral larvae to either the threshold concentration (3.5 ng/ml) or to a 10-fold elevated concentration (35 ng/ml) of the bacterial TBP-containing extract resulted in significant changes in the expression levels of eight out of 17 GOI in Assay 1 (*p<0.05*, Kruskal–Wallis test followed by multiple comparison of mean ranks; Figure 2A). These eight GOI (*Apextrin*, *NF-kB*, *AP-1/cFos*, *AP-1/cJun*, *CTL-1*, *MEKK-1*, *TIR-1* and *TRAF-6*) showed significant (*p<0.05*) differences in expression levels after exposure to the high concentration treatment compared to the control (Figure 2A). Following exposure to the threshold concentration, the genes, *Apextrin*, *NF-kB*, *AP-1/cFos*, *CTL-1*, *MEKK-1* and *TIR-1*, continued to exhibit significant (*p<0.05*) differences in expression levels.

### Assay 2

As for Assay 1, twelve hours post exposure, the expression levels of 16 out of 25 GOI in Assay 2 changed significantly (*p<0.05*, Kruskal–Wallis followed by multiple comparison of mean ranks; Figure 2B). Exposure to the high concentration treatment resulted in 16 GOI (*amilCP*, *amilCFP*, *amilGFP*, *amilRFP*, *Colorless GFP-like*, *GABA/GAT-3*, *GABA/unc-47*, *GABA/GAT-2*, *cAMP/ATF6*, *cAMP/ATF4*, *Actin*, *Pha-C3*, *LRRC59*, *Amgalaxin-like-1*, *SCRIP2* and *SCRIP3*) showing significant differences (*p<0.05*) in expression levels compared to the control (Figure 2B). Significant (*p<0.05*) differences in expression levels of eight of these genes (*amilCFP*, *amilGFP*, *Colorless GFP-like*, *GABA/GAT-3*, *GABA/GAT-2*, *cAMP/ATF6*, *Actin* and *SCRIP2*) after exposure to the threshold concentration treatment were also observed.

## Discussion

The induction of larval metamorphosis in *A. millepora* is triggered by exposure to biofilms of *Pseudoalteromonas* strain J010 and extracts thereof. The metamorphic cue in this extract, which completely bypasses larval attachment, has been identified as TBP [7]. The present study revealed that notable and relatively abrupt changes of the larval body structure during metamorphosis correlated, at the transcriptomic level, with differences in gene expression profiles observed in 24 out of 42 selected genes of interest (GOI; *p<0.05*; Figure 2). Differences in gene expression were scored at both threshold and elevated concentrations of bacterial TBP-containing extract in both assays. Twenty four GOI were differentially expressed after exposure to the 10-fold elevated concentration treatment. Fourteen of these were significantly regulated at the threshold concentration. These observations suggested that of the 24 GOI, ten genes required a higher threshold concentration of the bacterial TBP-containing extract in order to be regulated in the early stages of larval metamorphosis. These findings are in accordance with previous studies reporting that larval metamorphosis is controlled by genes that respond to specific threshold concentrations and gradients required to elicit a response to an inductive cue [34]. The results obtained in our study indicate this may also be valid for larval metamorphosis of *A. millepora*.

Crude extracts from CCA and the synthetic GLW-amide neuropeptide were previously used as cues to induce settlement of *A. millepora* [15]–[17], [35]. While larvae exposed to CCA-extracts generally responded with attachment followed by metamorphosis, the GLW-amide neuropeptide induced immediate metamorphosis with variable and significantly lower levels of attachment (40 to 80%) [14], [16], [17], [35]. Following exposure to 4 µM GLW-amide neuropeptide competent *Acropora* spp. larvae, which are completely covered with cilia surrounded by microvilli, continued to move actively for a few minutes, then attached perpendicularly to the surface [36]. After 6–9 hours the attached larvae flatten on the surface and form six primary mesentery filaments [36]. Competent swimming larvae exposed to the bacterial TBP-containing extract maintained normal swimming behaviour in the water column for 6 hours. Competent larvae exposed to CCA elongate prior to exploration and attachment [16]. However, elongation was not observed in larvae exposed to the bacterial TBP-containing extract, instead they became rounded and started to spin (6–12 hours), after which they flattened into buoyant, non-attached juveniles displaying obvious septal mesenteries radiating from the central mouth region. The change in swimming behaviour is possibly as a result of the change in the orientation, number or function of the cilia after exposure to the bacterial TBP-containing extract, indicating that induction of metamorphosis without surface exploration results in subsumption and/or degradation of cilia [37]. It was further observed that calcification was not initiated.

The up-regulation of *Amgalaxin–like-1* and *SCRiP3*, down-regulation in *SCRIP2* and *SCRiP-like* genes, and no changes in *CTL-2* were previously reported following 4 and 12 hours of exposure to GLW-amide neuropeptide [16], [17]. Our observation of similar expression of these genes, and the down-regulation of *CTL-1* and *Actin* (Figure 2), suggested that larvae underwent similar transcriptomic changes when exposed to the bacterial TBP-containing extract. SCRiP genes are found only in reef-building corals, suggesting that this gene family evolved following adaptations beneficial to the survival of this lineage [21]. Although their exact function in corals is not well understood, previous studies suggested that different SCRiPs may play distinct roles in the ontogenesis of corals, including metamorphosis [21]. Like SCRiPs, *Amgalaxin-like-1* encodes for a cysteine-rich, calcium-handling protein. It is expressed strongly in free swimming planulae and primary polyps during settlement and is thought to play a role in the initiation of calcification [20]. Our results (Figure 2B) support that *Amgalaxin-like-1* and the SCRiP genes are intimately involved in morphogenesis, but do not discount additional roles in attachment and calcification.

*Actin* encodes for one of the structural proteins involved in the development of cilia and was down-regulated following exposure to both treatments (Figure 2B). Down-regulation of *Actin* has been reported during initial attachment and subsequent metamorphosis of the bryozoan *Bugula neritina* [38] but was found to be up-regulated after 24 hours [38]. Rearrangement of cilia on the epidermal surfaces of *Acropora* spp. larvae following metamorphosis has previously been observed [36]. In the present study, larvae exposed to the bacterial TBP-containing extract lost their capacity to swim 6–12 hours after exposure. Following metamorphosis in the water column (Figure 1C), larvae most likely rearranged [36], internalized or discharged [37] their cilia, in line with the decreased expression levels of *Actin* observed.

A number of lectins were reported to be differentially expressed during settlement of *A. millepora* larvae [15]–[17]. *CTL-1* and *CTL-2*, C-type lectins which bind glycans in a calcium dependent manner, are thought to be involved in cell-cell adhesion and immunity responses [16]. The hemolytic lectins *HL-1*, *HL-2* and *HL-3* [16] along with the mannose-binding lectin, *millectin*, are also implicated in cell recognition and lysis for tissue remodelling during *A. millepora* metamorphosis as well as symbiont recognition [23]. Interestingly, after exposure to the bacterial TBP-containing extract, *CTL-1* was down regulated, whilst no change in expression levels were observed for the other lectins, correlating with no or very little attachment (0–2%) of metamorphosed larvae. These results suggested that lectins are not involved in morphogenesis, but are most likely expressed during larval exploration of substrates prior to attachment. Likewise the expression levels of the galactose binding lectins *Lectin/nemgal R1, B_1_* and-*B_2_*, which mediate symbiont engagement and maintenance in zooxanthellae–coral symbiosis [15], [39], did not change indicating that metamorphosis induced by the bacterial TBP-containing extract may bypass a number of essential ontogenetic processes.

The fluorescence-protein related genes (*amilCP*, *amilCFP*, *amilGFP*, *amilRFP* and *Colorless GFP-like*) were significantly (*p<0.05*) down-regulated following exposure to the TBP-containing bacterial extract of *Pseudoalteromonas* J010 (Figure 2B). Moderate down-regulation of GFP-like proteins in larvae of *A. millepora* was previously reported [29] during settlement for both green and red fluorescent protein transcripts. It has been suggested that the down-regulation of coral fluorescence genes is in response to changes in light exposure during normal settlement of coral larvae [29]. During the swimming stage, coral larvae are exposed to high light intensity in the upper water column. In the presence of a settlement cue, larvae migrated into the deeper water column where they attached to substrates in cryptic locations with inherently lower light intensity and subsequently metamorphosed. However, metamorphosed larvae incubated with the bacterial TBP-containing extract remained positively buoyant, indicating that fluorescence-protein related genes respond to the chemical cue, and that their down-regulation is coupled to metamorphosis rather than to changes in light intensity. In support of this observation, coral embryos raised in darkness showed relatively normal levels and distribution of mRNAs encoding GFP-like proteins [29]. It is therefore plausible that the reduced transcription of GFP homologues observed after exposure to the bacterial TBP-containing extract represents a regulatory mechanism enacted by the coral larvae to prevent wasteful allocation of resources (amino acids) for the production of non-essential proteins during metamorphosis rather than a response to reduced light intensity.

*Apextrin*, an extracellular protein that contains a membrane attack complex/perforin domain (MAC/PF), is a component of the complement cascade providing an alternate pathway to the production of *NF-κB* [19]. It is involved in apical cell-adhesion [40] and has been shown to be up-regulated during metamorphosis in sea urchins [40], [41] and settlement in *A. millepora* [17], [19], where it is located in ectodermal cells preceding metamorphosis. In this study, the *Apextrin* mRNA transcription was down-regulated in the presence of the bacterial TBP-containing extract (Figure 2A) resulting in no attachment, suggesting involvement in surface adhesion but not morphogenesis. This down-regulation is possibly another energy-saving mechanism to prevent wasteful allocation of resources in metamorphosed larvae that fail to attach. Further studies are necessary to characterise the regulation of *Apextrin* in response to the bacterial TBP-containing extract over a shorter timeframe and prior to the onset of larval morphological changes. This will assist in determining whether down-regulation of surface adhesion genes is involved in development of floating, non-attached, metamorphosed larvae.

The cnidarian complement *C3*, *Bf* and the *TX-60A* genes all contain a MAC/PF domain [19], [42]. Five genes: two *C3*, two factor B (*Bf*), and one mannan-binding protein-associated serine protease (MASP) comprising the complement system in cnidaria, are expressed in the tentacles, pharynx, and mesentery in an endoderm-specific manner [42]. The complement *C3* is initially expressed strongly in the endoderm of the planula as it elongates, while post-settlement expression is limited to the endoderm of the polyp as it rises from the calcifying platform at its base [19]. The venom gene *TX-60A* is expressed in the gland cells of cnidaria [19]. Our study has found that the expression of *C3*, *Bf* and *TX-60A* genes did not change significantly upon exposure to the TBP-containing extract, neither were they down-regulated, correlating with the larvae bypassing the processes of elongation, attachment and calcification, and indicating that they play a minor, if any, role in metamorphosis.

The amino-acid, γ-aminobutyric acid (GABA) and GABA-mimetic molecules, which act as inhibitory transmitters at invertebrate synapses, induced settlement of several marine invertebrate larvae, including the abalone *Haliotis rufescens* [43]–[45], and significantly increased the rate of metamorphosis in sea urchin [46]. Although the involvement of GABA transporters in coral larval settlement is not entirely clear, it has been postulated that these genes play a regulatory role in the cAMP dependent protein kinase-A phosphorylation involved in the movement of cilia [47] and larval exploration. Three of the four GABA transporter genes (*GABA/GAT-3*, *GABA/unc-47* and *GABA/GAT-2*) were significantly up-regulated in the high concentration treatment (Figure 2B). This up-regulation correlated with a cessation in swimming behaviour, as seen in the response of *Katharina tunicate* larvae to GABA at a concentration of 1 µM [48]. Interestingly, the cessation of swimming was followed by rapid settlement without any metamorphosis in the absence of CCA [48], indicating that GABA is not involved in metamorphosis itself. However, in acroporid coral larvae exposed to the bacterial TBP-containing extract, swimming decreased drastically and metamorphosis occurred in the water column without settlement. Grasso et al. [16] reported that elongation and searching behaviour of *A. millepora* prior to attachment to the substrate were responses to CCA, but not to GLW-amide. Our study showed that the bacterial TBP-containing extract did not initiate any of the above responses, rather it bypassed these processes and resulted in metamorphosis with no attachment.

NADH ubiquinone oxidoreductase is the first of three energy-transducing enzymes in the mitochondrial electron transport chain and a major source of reactive oxygen species in mitochondria and a significant contributor to cellular oxidative stress [49]. It is a highly regulated metabolic gene that was found to be involved in larval settlement and metamorphosis of *Hydroides elegans* [50]. This gene, changed following induction by CCA in *Haliotis asinine* [51], during thermal stress of the coral *Montastraea faveolata* [22] and following exposure of *A. millepora* larvae to CCA [17]. Surprisingly, the expression level of NADH ubiquinone oxidoreductase did not change between free swimming coral larvae and those that metamorphosed after exposure to the bacterial TBP-containing extract indicating its function is primarily in general cellular metabolism.

The cAMP-dependent protein kinase A (*HePKAc*) mediates larval development and settlement in *Hydroides elegans* [52] with increased expression patterns during larval development and decreased expression patterns in the adult stage [52]. In *A. millepora* larvae exposed to the bacterial TBP-containing extract, the closely related gene *Pha-C3* was up-regulated confirming its role in morphogenesis, however, this does not rule out further function in cell-adhesion and/or motility.

*A millepora* genes closely related to vertebrate counterparts within the classic transmembrane Toll/TLR pathways, have been described previously [19]. The expression levels of these related genes varied during metamorphosis induced by the bacterial TBP-containing extract; *LRRC59*, *TIR-1* and *MEKK-1* were up-regulated, no changes were observed in the expression of *ERK2* or *MAPK-p38*, and *TRAF-6* and *NF-κB* were both down-regulated. Based on this expression profile we postulate that the MAPK pathway rather than the *NF-κB* pathway is the dominant signalling pathway utilised during morphogenesis by *A. millepora*. It should be noted, however, that the Toll/TLR signalling pathways have not been fully elucidated in corals.

The MAPK signalling pathway provides a further link between the Toll/TLR and TGF-b (transforming growth factor-beta)/BMP (bone morphogenic protein) pathways in vertebrates [19], [53]. It transmits signals from receptor to nucleus via the cAMP dependent activating transcription factor (ATF) genes, which are known to have pleiotropic functions in later stages of development and metamorphosis of drosophila larvae [54]. *AP-1cFos* was up-regulated and *AP-1cJun*, *cAMP*/*ATF4* (regulates amino acid metabolism and resistance to oxidative stress) and *cAMP*/*ATF6* (mediates response to endoplasmic reticulum stress) were down-regulated in response to the TBP-containing extract whereas *cAMP*/*ATF2* (mediated by both TGF-b and MAPK pathways) and *ATF4/5* (*ATF4*-like) showed no significant change. A recent study has shown that *AP-1cJun*, *ATF2*, *ATF4* and *ATF6* were regulated following exposure of *A. millepora* larvae to CCA, GLW-amide neuropeptide and temperature stress [17]. It should be noted that ATF and AP-1-mediated regulation of processes such as cellular proliferation, differentiation and transformation is influenced by a number of other signalling pathways and it is not clear from the current study exactly which genes are involved specifically in metamorphosis only. While the components of the different pathways are represented in the cnidarian database [19], [55], the coral signalling pathways have not yet been elucidated and all the available information relates strictly to vertebrates.

Septins are proteins that participate in the establishment and maintenance of asymmetry during cell morphogenesis by creating a barrier to diffusion of other factions along a membrane [56]. They have been implicated in larval attachment and metamorphosis of the polychaete *Hydroides elegans* [50]. No significant changes in gene expression for either *Septin-1* or *Septin-7*, at either concentration of the bacterial TBP-containing extract, were observed in the metamorphosed larvae. It is feasible that once differentiation has transpired the expression level is returned to a basal level. Further studies over a shorter timeframe are necessary to characterise the regulation of these septins in response to the bacterial TBP-containing extract and to determine whether up-regulation of these genes occurs immediately after exposure to the cue but prior to any observable cellular restructuring.

### Conclusion

The GOI examined in this study were chosen because they encode key proteins reported to be involved in metabolism and settlement of invertebrate larvae. Moreover, the bacterial TBP-containing extract used in this study was considered a unique tool to specifically induce the metamorphic stage in *A. millepora* larvae without attachment. This specificity is fundamental to monitor the performance and regulation of genes exclusively involved in the process of metamorphosis without potential inclusion of mRNA readings from larvae undergoing settlement.

*Actin* and *Amgalaxin-like-1* responded to the bacterial TBP-containing extract in a similar way to the metamorphic cue GLW-amide neuropeptide, and coincided with decreased larval swimming behaviour suggesting these genes are implicated in cilia development and larval swimming capability and not attachment. Likewise *Apextrin* and the lectins investigated in this study are most likely involved in the attachment process of settlement (cell-adhesion) and not metamorphosis. Furthermore, the Toll/TLR pathways genes investigated in this studied indicated that the MAPK pathway rather than the NF-κB pathway is the dominant signalling pathway utilised during morphogenesis by *A. millepora*.

While the emphasis of this study was to understand the process of metamorphosis occurring between 6 and 12 hours following initial exposure to the bacterial TBP-containing extract, we conclude that some of the measurable changes in gene regulation may relate to other processes and are not exclusively in response to the bacterial TBP-containing extract. Shorter exposure times to metamorphic cues and a higher sampling frequency of larvae prior to attachment and/or metamorphosis are required to detect early changes and will improve our understanding of gene regulation in coral larval metamorphosis. Furthermore, a comparison of the larval response during metamorphosis to more than one settlement cue will be crucial to identify key genes involved in coral ontogensis in future studies. The ability to examine gene expression in the different life stages of corals using the low cost, accurate, ecologically relevant, highly reproducible RT-qPCR assays developed in this study has enabled us to begin decoupling the process of attachment (specifically elongation and calcification) from metamorphosis.

## Materials and Methods

### Ethics Statement

Authority to enter the Ningaloo Reef Marine Park to collect and re-attach the coral colonies used in this study was granted by the Government of West Australia Department of Environment and Conservation (permits CE002767 and SF007369). No ethical approval was required for any of the experimental research described here.

### Sample collections and larval maintenance

Five colonies of *A. millepora* were collected from Coral Bay, Ningaloo Reef (23°08′S, 113°45′E) in Western Australia on 5^th^ April 2010, four days prior to the predicted mass spawning event. Coral colonies were kept on the reef tied to racks at a depth of 2–3 m. For gamete collection colonies were transferred each night, approximately 2 hours prior to the predicted spawning time 21:00, to 60 l containers filled with 1 µm-filtered seawater. The colonies were returned to the reef 2 hours after the predicted spawning time. Following spawning (day 4, 8^th^ April 2010 at 21:00), gametes from all five colonies were cross-fertilised. After the first mitotic division embryos were transferred into three 300 l vessels containing 1 µm-filtered seawater at 23±1°C for larval development. Larvae were maintained in these tanks with a filtered seawater flow rate of approximately 0.5 l/min (in an outdoor shaded area) for 12 days before exposure to metamorphic cues.

### Growth conditions and extraction of metamorphosis-inducing bacteria

Growth conditions of *Pseudoalteromonas* strain J010 and the downstream procedures of extraction and purification of the chemical inducer of larval metamorphosis, TBP, were adopted from Tebben et al. [7]. *Pseudoalteromonas* strain J010 was spread onto 60 agar plates (100% marine agar) and cultivated at 27–29°C for 48 hours. Subsequently, 3.5 g of bacterial biofilm was collected from the surface of the agar using a sterile spreader and extracted with 50 ml ethanol (0.07 g/ml). Since TBP was present in a single fraction of J010, and there were no significant differences in the metamorphosis ratio between crude ethanol extracts of the bacterial strain and trials employing TBP [7], the crude extract was used directly.

### Exposure of larvae to metamorphic cues

The minimum threshold concentration of the crude extract required to trigger larval metamorphosis after 12 hours was determined in a dose response experiment as 3.5 ng/ml. Gene expression patterns were measured at the threshold concentration (3.5 ng/ml) and at a 10-fold elevated concentration (35 ng/ml) prepared by dissolving 100 and 10 µl of evaporated extract in 200 ml of 1 µm-filtered seawater. Higher crude extract concentrations induced metamorphosis in less than 3 hours (data not shown). Each treatment received 200–300 *A. millepora* larvae. Six replicates were prepared for each experimental treatment including a control (no extract) and incubated for 12 hours at room temperature (27–29°C). Subsequently, metamorphosed and swimming larvae were collected by gentle filtration using a 60 µm mesh and preserved in RNAlater (Qiagen) according to the manufacturer's recommendations. Samples were transported at −150°C in a dry shipper and stored at −80°C until required for analysis.

### Gene selection and multiplex design

Two multiplex RT–qPCR assays capable of profiling gene expression levels from a total of 52 selected genes in *A. millepora* (47 GOI; 5 RG) were developed (Tables S1 and S2). The vast majority of GOI were selected for their putative role(s) in the processes of settlement and metamorphosis whilst some of them are also known to be involved in the coral's innate immune defence pathways against pathogens and environmental stressors during ontogenesis [15]–[17], [19]–[21]. Twenty four genes were selected to build a multiplexed RT-qPCR assay to investigate the variation in immunity-related genes potentially involved in ontogenesis of *A. millepora*. Of these 24 genes, 20 represented GOI while four genes served as RG [24], [26] (Table S1). The aforementioned plus an additional RG [57] were combined with 27 selected putative GOI involved specifically in larval settlement and/or metamorphosis to produce a second multiplexed RT-qPCR assay (Table S2). The specific function and expression of the selected genes based on previous studies are summarised in Table 1.

Gene identities were confirmed via the BLASTp algorithm against public databases (http://blast.ncbi.nlm.nih.gov), and sequences were uploaded into the express multiplex design module (EMDM) of the Beckman Coulter GenomeLab™ GeXP eXpress Profiler software. For each multiplex reaction, the kanamycin (*Kan* ^r^) gene was assigned as the internal control gene (positive/calibration control) and the minimum separation size of 10 and 7 base pairs (bp) for each assay, respectively, were assigned as fixed distances among amplification products. Single gene PCR product sizes, excluding the universal tags, ranged from 100 to 380 bp. Annealing temperatures were optimized by the software given the combination of primers (Tables S1 and S2).

### Messenger RNA extraction, cDNA preparation, PCR amplifications & electrophoresis

Larval mRNA was extracted using Dynabeads (Invitrogen) according to the manufacturer's instructions, and diluted to 3.2 ng/µl. Reverse transcription of 6.4 ng mRNA to cDNA was initiated in a 10 µl reaction containing 2 µl reverse transcription buffer (5×), 0.5 µl reverse transcriptase (20 U/µl), 1 µl of reverse primer multiplex and 2.5 µl *Kan^r^* RNA. One negative template (T–) and one negative Reverse Transcriptase (RT–) (*Kan^r^* only) control were used to test for cDNA and RNA contaminants, respectively, according to the GenomeLab™ GeXP manual (a detailed description for this procedure is reported in [26]). The reaction was cycled through the following temperatures: 1 min at 48°C, 60 min at 42°C, 5 min at 95°C, finishing with 4°C. PCR amplifications were initiated in a 10 µl reaction volume containing 2 µl PCR buffer (5×), 2 µl of 25 mM MgCl_2_, 1 µl custom PCR Fwd mix, 0.35 µl DNA polymerase and 4.65 µl cDNA. The reaction was cycled through the following temperatures: 15 min at 95°C, followed by 30 sec at 94°C, 30 sec at 55°C, 1 min at 68.5°C, 5 min at 95°C for 34 cycles, finishing with 4°C. All the reagents for cDNA and PCR experiments were acquired from Beckman Coulter (Australia, Gladesville). For Assay 1, each forward primer concentration was 0.2 µM. The concentration of each reverse primer was 0.5 µM except *Rps7* which was lowered to 0.03 µM. For Assay 2, the concentration of each forward primer was 0.2 µM and for each reverse primer was 0.5 µM except for *Rps7* 0.004 µM, *Actin* 0.0625 µM, *AmilRFP* 0.05 µM and *AmilCP* 0.0625 µM. The modification of the oligo concentrations was necessary during the optimization process in order to include the expression level of the corresponding genes in the optimal detection range of the analysis algorithm. Diluted PCR products (1∶60 for Assay 1 and 1∶20 for Assay 2) were analysed individually on an automated capillary electrophoresis sequencer CEQ™ 8800 Genetic Analysis System (Beckman-Coulter, Fullerton, CA, USA) as follows: diluted PCR products (1 µl) were loaded into a 96-well plate with 0.2 µl of DNA size standard (400 bp; Beckman-Coulter) and 38.8 µl of GenomeLab Sample loading solution (SLS).

### Data visualization, filtering & statistical analysis

Electropherograms were visualized, filtered and matrices generated for further analyses following the GenomeLab™ GeXP manual [26]. An expression stability measure for all the genes in both assays was performed using the GeNorm program (http://medgen.ugent.be/genorm/) according to Vandesompele et al. [33] and the most stable pair of genes used to normalize the gene expression levels of the GOI in Microsoft Excel 2007 using the Geomean function. For data analysis, only samples with at least two technical repeats and coefficient of variation (CV) lower than 25% were considered. Statistical analysis was conducted using Kruskal–Wallis (non-parametric one way analysis of variance by ranks) followed by multiple comparison of mean ranks for all groups performed using STATISTICA version 10.0 (StatSoft, Tulsa, OK).

## Supporting Information

Table S1 **List of genes employed in the multiplexed RT-qPCR assay 1.**
(PDF)

Table S2 **List of genes employed in the multiplexed RT-qPCR assay 2.**
(PDF)
