# Supplementary material for: Using Bacterial Extract along with Differential Gene Expression in Acropora millepora Larvae to Decouple the Processes of Attachment and Metamorphosis
Source: PLoS One. 2012 May 24;7(5):e37774. doi: 10.1371/journal.pone.0037774 (PMC3359992; doi:10.1371/journal.pone.0037774)
Supplement: Table S1 — List of genes employed in the multiplexed RT-qPCR assay 1. (PDF) [file pone.0037774.s001.pdf]

Table S1. List of genes employed in the multiplexed RT-qPCR assay 1.

Protein name	Acronym	Accession number	Product length	Oligo sequences[a]	Function	References
**Reference genes**						
Glyceraldehyde 3 phosphate dehydrogenase	*Gapdh*	EZ026309	187	F: AGGTGGAGCCAAGAAAGTCA<br>R: TTAGCTAGAGGAGCCAGGCA	Housekeeping gene of demonstrated stable expression level	[24,26]
Ribosomal protein S7	*Rps7*	EZ031290	197	F: CAGGCATGCTTACAACCAAA<br>R: TCAACCTCCTTTGCTCCAGT		
Ribosomal protein L9	*Rpl9*	EZ026324	292	F: CGTGTAAACGTGTGGTTTGC<br>R: TTTGACACCTGAATTCGCAC		
Unknown transcript	*Ctg1913*	EZ040581	280	F: GATTTAACCACCGGCAGTGT<br>R: ATGGTAGGGAGGAGGCTGTT		
**Genes of interest**						
Toll interleukin receptor	*TIR-1*	EF090256	137	F: AAAGCCGCAGTCATCAGTTT<br>R: GAAATTGGCGTTGAATTCGT	Toll/TLR pathway	[19]
TNF receptor associated factor 6	*TRAF-6*	DY583189	127	F: TGATGAATGTCCTTTCGCAG<br>R: ACATGCTTTGCAAGCTGATG		
Mitogen-activated protein kinase/ERK kinase kinase-1	*MEKK-1*	DY581208<br>DY581138<br>DY582675	117	F: CTGCGGATATTTGGTCCTGT<br>R: TTTCTTTGTCGGTTGATCCC		
Extracellular signal regulated kinases	*ERK-2*	EZ025389	217	F: CCAAAGGTTACAGCAAGGCT<br>R: TGCGTGCCTTTTCATTCATA		
P38 mitogen-activated protein kinases.	*MAPK-p38*	EZ031759	237	F: AAAATCAGCAGTGAATCCGC<br>R: TCGGGGTCTGAATACGTAGC		
Part of AP-1 early response transcription factor	*AP-1/cFos*	EZ016042	177	F: CTGGAAAGAGAATTGCTGGC<br>R: GACGATTGCACTTCGGACTT		
Part of AP-1 early response transcription factor	*AP-1/cJun*	EZ020860	366	F: TCGATCGAAGGGACAGTTCT<br>R: GTGCTAGTTGCGGTGTTCAA		
c-AMP Activating transcription factor 4	*ATF4/5*	DY577805	247	F: GGCCAGAACGTATCACCAAT<br>R: TCTTCGAAATCAAACCCCTG		
Nuclear factor kappa B	*NF-kB*	G0002043<br>DY582971<br>DY580118<br>G0000491	152	F: GATGTTGCAGGCTCAGTTCA<br>R: CTCATATGCAGGTTGGTGGA		
TRIF-related adaptor molecule	*TRAM*	EZ047194	157	F: AAGCTAACGGCTCACCAAGA<br>R: TGTGCCATGCACAAGAAAAT	IFN pathway	[19]
Apical extracellular protein	*Apextrin*	EF091848	352	F: GGATTCGTACCAAAAAGGCA<br>R: GAGGGGTCTGATATGGGGTT	MAC/PF - membrane attack complex/perforin domain.	[19]
60 kDa proteinaceous toxin	*Tx60-A*	DY579588	207	F: TACTGCCCTTGAGGTTTGCT<br>R: CTGAAAATCCCGCTGACTGT		
Complement factor B precursor	*Bf*	GO001635	227	F: TTATCCATCCCGACGCTAAC<br>R: AGGATCATCTTTTCCTGCGA	Alternative pathway of complement activation with 6e-77 on 30% coverage to *N. vectensis* (AB450042).	[42]
Complement C3	*C3*	EF090257	167	F: CCGCTACACGCTAGACAACA<br>R: CCGCAGAGTCGATGTACAAA	Alternative pathway of complement activation with 0e-value on 87% coverage to *N. vectensis* (AB450038).	[42]
Mannose-binding lectin	*millectin*	EU717895	257	F: AGCGAGTATCCACAACACCC<br>R: GGCTTTTTCGATGTTTTCCA	Innate immune system cell surface recognition receptors.	[15,16,23]
C type lectin	*CTL-1*	GO001638	312	F: GGGTTGTGTACAACGGCTTT<br>R: CTTTCCATTCGGTTCTCCTG	Innate immune system cell surface recognition receptors.	[15,16]
C type lectin	*CTL-2*	GS01UH10	267	F: CAGGTCTGGATCGGACTCAT<br>R: CATGTCCAGTGGTTGTACGC		
Hemolytic lectin-1	*HL-1*	EU863776<br>EU863777	335	F: TTCGCTCCAGAGGGAAACTA<br>R: GCAGAAATGCCTTTGGTTGT	Innate immune system cell surface recognition receptors, member of the CEL-III lectins.	[15,16]
Hemolytic lectin-2	*HL-2*	EU863776	302	F: AACAGTTGAGATAACCGCCG<br>R: TTGATTCCTGGTGCATTTGA		
Hemolytic lectin-3	*HL-3*	EU863777	379	F: TTCTGGAGATTGGGTAACGC<br>R: TCGTTCTCAGCGTGTTGTTC		

[a] Forward (AGGTGACACTATAGAATA) and reverse (GTACGACTCACTATAGGGA) fluorescent universal tags, omitted from the oligo sequences in the table, were added at the 5’ end of all primers.
