# Supplementary material for: Using Bacterial Extract along with Differential Gene Expression in Acropora millepora Larvae to Decouple the Processes of Attachment and Metamorphosis
Source: PLoS One. 2012 May 24;7(5):e37774. doi: 10.1371/journal.pone.0037774 (PMC3359992; doi:10.1371/journal.pone.0037774)
Supplement: Table S2 — List of genes employed in the multiplexed RT-qPCR assay 2. (PDF) [file pone.0037774.s002.pdf]

Table S2. List of genes employed in the multiplexed RT-qPCR assay 2.

Protein name	Acronym	Accession number	Product length	Oligo sequences[a]	Function	References
**Reference genes**						
Glyceraldehyde 3 phosphate dehydrogenase	*Gapdh*	EZ026309	187	F: AGGTGGAGCCAAGAAAGTCA<br>R: TTAGCTAGAGGAGCCAGGCA	Housekeeping gene of demonstrated stable expression level	[24,26]
Ribosomal protein S7	*Rps7*	EZ031290	197	F: CAGGCATGCTTACAACCAAA<br>R: TCAACCTCCTTTGCTCCAGT		
Ribosomal protein L9	*Rpl9*	EZ026324	292	F: CGTGTAAACGTGTGGTTTGC<br>R: TTTGACACCTGAATTCGCAC		
Unknown transcript	*Ctg1913*	EZ040581	280	F: GATTTAACCACCGGCAGTGT<br>R: ATGGTAGGGAGGAGGCTGTT		
Microtubule affinity-regulating kinase 3.	*Mark3*	EZ011755	162	F: GAGCAGAAGACCGAAACAGC<br>R: AGGCTGCTTAGAGGAGGGTC	Housekeeping gene of demonstrated stable expression level	[57]
**Genes of interest**						
GTPases hydrolase enzyme	*Septin-1*	EZ027858	137	F: TTAACCATTGGTGGCATGAA<br>R: GTTCCGGTTCTTGTTTTGGA	Cell division and cytokinesis.	[50]
GTPases hydrolase enzyme	*Septin-7*	EZ044856	157	F: TTTGCGAAGTTTTAGAGCGG<br>R: CCTTTCTTGACGCTCTTTCG		
Leucine-rich repeat-containing protein 59	*LRRC59*	EZ012188	182	F: CGGCATTACCTAAAGCTAGGG<br>R: AGATTGCCAAACTCCTGTGG	Toll/TLR pathway with 3e-50 on 95% coverage to *Hydra magnipapillata* (XP_002165534.1).	[58]
Cyclic AMP-dependent transcription factor ATF-2	*cAMP/ATF2*	EZ023176	202	F: TGCTAGCAGTCCTCCCTCAT<br>R: ACATGGTTTCCTGGACTTGG	Classic Toll/TLR pathway.	[19,44,52,59]
Cyclic AMP-dependent transcription factor ATF-4	*cAMP/ATF4*	EZ013045	177	F: CAAGTGACCCTCCTTCTCCA<br>R: AGAGACACATTCCCCAGGTG		
Cyclic AMP-dependent transcription factor ATF-6	*cAMP/ATF6*	EZ023811	285	F: GACGAGTTGCTTTGGGAAAA<br>R: TGCACTGGTGAAGTGTCTCC		
Tyrosine aminotransferase	*tyr-amTase*	GH999932	192	F: TCTTTCCATCGGTGATCCAT<br>R: AATGTTCAGCTGCGATAGCC	Tyrosin aminotransferase with 7e-37 on 85% coverage to *N. vectensis* (XP_001632576.1)	[51,60]
Sodium and chloride-dependent γ-aminobutyric acid transporter 2	*GABA/GAT-2*	EZ004523	152	F: TGGCCTGGGTGATCTGTTAC<br>R: CCTCGAATCAATAGTGCCGT	Neurotransmitter (facilitates communication between neurons).	[43-45]
Sodium and chloride-dependent γ-aminobutyric acid transporter 3	*GABA/GAT-3*	EZ026165	207	F: CCAGGGAGTTGAAGTGGAAA<br>R: AAAGCCTTCGATGCCTACAA		
Vesicular γ-aminobutyric acid transporter	*GABA/unc-47*	EZ005918	212	F: TTACCAACGGTTGAGTTGAGC<br>R: TTCGTATTGGTCCTTCAGGC		
Sodium and chloride-dependent γ-aminobutyric acid transporter ine	*GABA/ine*	EZ004722	217	F: CCAAAATGCGTGGGATACAG<br>R: CCTTCCTTCGTTTTTGTGGA		
NADH-ubiquinone oxidoreductase	*NADH-ubiq*	DY585529	222	F: ACTTTGGAAGGCTTTCCCTC<br>R: AACAGGCCAGACCAAATGTC	First enzyme of the mitochondrial electron transport chain, associated with proton translocation across the membrane	[51]
Galactose binding lectin domain, nematogalectin-related	*Lectin/nemgalR1*	EU863779	142	F: TGAAATCAGATGCACCAAGC<br>R: TCGTGCCCGTTTATAACCTC	Innate immune system cell surface recognition receptors.	[15,16]
Galactose binding lectin domain, nematogalectin-related	*Lectin/nemgal*B_1	EU863780	227	F: GCGATGCGATGCTTATTACA<br>R: TCCAATCTTCGGCTTACTGG		
Galactose binding lectin domain, nematogalectin-related	*Lectin/nemgal*B_2	EU863778	270	F: ACATGTCCCAAGATTCCAGC<br>R: CCTCCTTCTTTCAGCACGTC		
Serine/Threonine Kinases family DC2	*Pha-C3*	EZ023480	232	F: GATATGCTTCCCTTCACCCA<br>R: GGCTGACACAGAGGGTTCTC	cAMP-dependent protein kinase A with 4e-113 on 90% coverage to *Hydroides elegans* (AB232160).	[52]
Green fluorescent protein	*amilGFP*	AY646067	147	F: TAGTGATCATCAAGCCTGCG<br>R: AGGCATGTCTGCAGGGTACT	Fluorescent proteins account for visible coral colour other except brown (photosynthetic pigments of algal symbionts).	[25,29,61]
Cyan fluorescent protein	*amilCFP*	AY646070	245	F: GAGGAGTTGCTACAGCCAGC<br>R: CCACCTCCTTCGAGCATAAG		
Red fluorescent protein	*amilRFP*	AY646073	172	F: CCATCCTGCGAGAAAATCAT<br>R: CGGGTCAGACTTTGCTTTGT		
Blue chromoprotein	*amilCP*	AY646075	239	F: ACGGAAGCATACCATTCACC<br>R: GGTCCATTGGGAGGAAAGTT		
Colourless green fluorescent like protein	*Colourless GFP-like*	AY650288	265	F: CAGCTGGATACACATGGCAC<br>R: CCCCTTTCAATATCCCCTGT		
Cysteine-rich protein	*Amgalaxin-like-1*	HM163216	167	F: GTGGCAGGATTCGCTACATT<br>R: CCGAAACAGCACTTGTGAGA	Galaxin-related gene.	[20]
Small cysteine-rich protein	*SCRiP-like*	DY587111	250	F: AGCGACCATAAGTTCGCAAG<br>R: CATCACTTGTGCTAAGGGCA	Coral development and biomineralization	[21]
Small cysteine-rich protein	*SCRiP2*	BK006535	255	F: TTGATTTTACTGCTGGGCCT<br>R: GGATAGCAGCAGCAGTGGTT		
Small cysteine-rich protein	*SCRiP3*	BK006536	260	F: GTTCGCAAGGGTTCAATCTC<br>R: ATTCCTTTGGGCATCACTTG		
Some actin	*Actin*	DY579126	298	F: ATGAAGCTCAAAGCAAGCGT<br>R: ACAGGGACAATACAGCCTGG	Structural filament protein.	[38,62]
Type I heterodimeric integral membrane glycoprotein	*Integrin-alpha1*	EU239371	303	F: TCAAGTTTTCACCCGGTAGG<br>R: AGGACCATTATTACGCACCG	Cell-cell and cell-extracellular matrix adhesion.	[64]

[a] Forward (AGGTGACACTATAGAATA) and reverse (GTACGACTCACTATAGGGA) fluorescent universal tags, omitted from the oligo sequences in the table, were added at the 5’ end of all primers.
